# Notes and News

**Published:** 1897-12-11

**Authors:** 


### NOTES AND NEWS.

(Collected and Communicated.)

The Grocers' Company have made a grant of £250 to St. Thomas's Hospital.

The Queen has presented the Jubilee Medal to Sir Henry Burdett, K.C.B., as an additional recognition of his public services.

The Prince of Wales has contributed £5 towards Alderman Treloar's fund for providing 5,000 crippled children with Christmas hampers.

The Countess of Morley has laid the foundation-stone of the new eye infirmary for Plymouth. Thirty patients are to be accommodated in four wards. The total outlay is estimated at £14,558.

An interesting series of books entitled "Masters of Medicine" is being brought out by Messrs. Fisher Unwin, under the editorship of Mr. Ernest Hart. The two first volumes, devoted to the lives of John Hunter and William Harvey, are ready, whilst that on Jenner —written by Mr. Hart himself—and many others, are in preparation.

The late Sir William Mackinnon, hon. surgeon to the Queen, and formerly Director-General of the Army Medical Department, has left money to found scholarships and prizes in science at the University of Edinburgh, and consigns the residue of his property to the Royal Society for the furtherance of Science. Pathology is the only branch of medicine or surgery that is included in his benefactions.

The Radcliffe Infirmary has begun to move, and we are glad to learn that an old block—the Hakewell—which, as one of the governors pointed out at a recent meeting, had been "more threatened and condemned than any other building he ever remembered" is to be demolished. In place of it is to be erected a new operating theatre, in substitution for the present one, which is most inconveniently situated.

The new wing to be added to the Ipswich and East Suffolk Hospital as a Jubilee Commemoration has been commenced. Two wards, with twelve beds in each, will be provided. These wards will communicate with the existing building by wide covered corridors. Simultaneously with the work on the new buildings improvements in the hospital as it now stands will be carried on, so as to bring it more in a level with modern requirements.

The Duke of Connaught will take the chair at the Festival Dinner in aid of Saint Mark's Hospital, to be held at the Whitehall Rooms, on Monday, April 25th, 1898—St. Mark's Day.

The munificent sum of £1,500 has been received by the Prince of Wales's Hospital Fund from the Licensed Victuallers' Protection Society of London. This, we learn, is only a first instalment.

The Children's Committee of the Metropolitan Asylums Board have purchased 28 acres of land at Brentwood as a site for an ophthalmic school. The Board are about to enlarge the Convalescent Home at Leavesden Asylum in order to convert it into an isolation hospital.

The foundation-stone of the new Infirmary and Nurses' Home at Kingston has been laid by Miss Lucy Witten, daughter of the Chairman of the Board of Guardians. There will be accommodation for 132 beds in the new infirmary. On the ground floor are to be placed five wards containing from sixteen to two beds in each, and the usual offices and nurses' room. To the south there is to be a verandah. On the first and second floors will be five wards again, with the necessary offices. These floors will be provided with balconies above the verandah. The floors are to be of pitch pine blocks on a hollow brick fireproof foundation. Warm-air stoves and hot-water coils will constitute the warming apparatus. The cost of the institution is estimated at £18,000.

The Jubilee year continues to bring golden gifts to the hospitals. The latest good fortune comes from legacies under the will of the late Mrs. C. Armitage, of St. John's Wood. She has left to the National Consumption Hospital, St. Mary's, Guy's, the Middlesex, and Queen Charlotte's Hospitals each £4,000. The London Fever Hospital and the Hospital for Epilepsy and Paralysis are also to have large sums, which, if the residue permits, is to reach £4,000 in each instance. Then any surplus will go to the first-named hospitals. All these sums Mrs. Armitage bequeathed from her husband's estate. From the residue of her own estate the Hospital for Sick Children, the Paddington Green Children's Hospital, and the Hospital for Women and Children will in all probability receive about £5,000 each.

Lady Pilkington recently relaid the foundation-stone of a children's ward in the old Southport Infirmary in the corresponding ward in the new institution. The original ceremony was performed by her in 1885. It was a happy thought thus to rescue from oblivion an object to which so much interest attaches, and it is really to be regretted that such landmarks should be buried from view and frequently entirely lost sight of. At the British Home for Incurables a much happier proceeding has been adopted. The foundation-stone is preserved for ever in full view of every visitor to the establishment, forming part of a handsome column at the base of the main staircase. Its inscription bears the history of the foundation of the home, and a remembrance of the interesting incidents of the ceremony in connection. So excellent a precedent might well be followed.

